# Identification, expression, and functional analysis of *Hsf* and *Hsp20* gene families in *Brachypodium distachyon* under heat stress

**DOI:** 10.7717/peerj.12267

**Published:** 2021-10-01

**Authors:** Na Li, Min Jiang, Peng Li, Xiwen Li

**Affiliations:** 1 Shanghai Key Laboratory of Plant Functional Genomics and Resources, Shanghai Chenshan Botanical Garden, Shanghai, China; 2 College of Life Sciences, Shanghai Normal University, Shanghai, China; 3 Ministry of Education Key Laboratory for Biodiversity Science and Ecological Engineering, Institute of Eco-Chongming (IEC), School of Life Sciences, Fudan University, Shanghai, China

**Keywords:** Heat shock protein, Heat shock factor, Evolution, Seed germination, *Brachypodium distachyon*, Correlation analysis

## Abstract

**Background:**

The heat shock factor (Hsf) and small heat shock protein (sHsp, also called Hsp20) complex has been identified as a primary component in the protection of plant cells from ubiquitous stresses, particularly heat stress. Our study aimed to characterize and analyze the *Hsf* and *Hsp* genes in *Brachypodium distachyon*, an annual temperate grass and model plant in cereal and grass studies.

**Results:**

We identified 24 *Hsf* and 18 *Hsp20* genes in *B. distachyon* and explored their evolution in gene organization, sequence features, chromosomal localization, and gene duplication. Our phylogenetic analysis showed that BdHsfs could be divided into three categories and BdHsp20s into ten subfamilies. Further analysis showed that the 3’UTR length of *BdHsp20* genes had a negative relationship with their expression under heat stress. Expression analyses indicated that *BdHsp20s* and *BdHsfs* were strongly and rapidly induced by high-temperature treatment. Additionally, we constructed a complex regulatory network based on their expression patterns under heat stress. Morphological analysis suggested that the overexpression of five *BdHsp20* genes enhanced the seed germination rate and decreased cell death under high temperatures.

**Conclusion:**

Ultimately, our study provided important evolutionary and functional characterizations for future research on the regulatory mechanisms of *BdHsp20s* and *BdHsfs* in herbaceous plants under environmental stress.

## Background

Although plants may appear inactive, they have actually evolved a series of countermeasures to respond to various extracellular stimuli. Therefore, plants are not completely passive under external stresses and will form some complex protective mechanisms for adaptation. Abiotic stress, such as high temperature, high salt, heavy metal pollution, and drought, threaten crops’ normal growth and development (Wang, Vinocur & Altman, 2003; Mittler, 2006). Global warming resulting from the greenhouse effect has made high temperatures a greater threat to plants and the main factor affecting plant productivity. It is well-known that heat shock factors (Hsfs) (Nover et al., 2001) and heat shock proteins (Hsps) (Lindquist & Craig, 1988) are initial response genes that can be rapidly induced to repsond to high temperatures. Under external stimuli, *Hsp* expression can be induced by *Hsfs* binding the heat shock elements (HSEs; nGAAnnTTCn or nTTCnnGAAn) that exist in multiple copies upstream of the *Hsp* genes (Giorno et al., 2010). *Hsp* acts as a molecular chaperone that prevents protein misfolding and eliminates non-natural protein aggregation.

Hsps are widespread in living organisms and generally fall into five groups according to their molecular weight: Hsp100, Hsp90, Hsp70, Hsp60, and Hsp20 (Marrs et al., 1993; Schirmer, Lindquist & Vierling, 1994; Krishna et al., 1995; Mayer & Bukau, 2005; Waters, 2013). Hsp20 is a structurally conserved small heat shock protein (sHsp) with a molecular weight of 15–42 KD. It consists of a variable N-terminal region, a conserved C-terminal region, and a C-terminal extension region (Kriehuber et al., 2010; Poulain, Gelly & Flatters, 2010; Waters, 2013). The N-terminal region sequences across different subfamilies vary greatly, while their sequences in the same subfamily show high similarity (Bondino, Valle & Have, 2012). In contrast, the C-terminal sequence contains a conserved a-crystallin domain (ACD), composed of approximately 90 amino acids, and can be divided into CRI and CRII regions with a β_6_-loop junction in the middle region. sHsps are the “first responders” in response to cellular stress. They are ATP-independent chaperones that do not change the protein activity themselves, but instead bind to denature-modified proteins of the same molecular weight to form stable compounds and prevent their aggregation (Hartl, Bracher & Hayer-Hartl, 2011). For example, LimHSP16.45 and OsHSP18.2 are molecular chaperones that enhance cell viability under stress (Mu et al., 2011; Kaur et al., 2015). Additionally, abiotic stresses other than high temperature, such as drought, low temperature, high salt, heavy metals, anaerobic stress, and UV-light, can also induce sHsp synthesis (Wang et al., 2004; Sun & Macrae, 2005).

Unlike Hsps, Hsfs usually contain five conserved domains: the DNA binding domain (DBD), hydrophobic oligomerization domain (HR-A/B), nuclear localization signal domain (NLS), nuclear export signal (NES), and C-terminal activation domain (CTAD), which is characterized by short peptide motifs (AHA motifs). Hsfs can be divided into three subfamilies based on the number of amino acid residues inserted between HR-A/B: Class A, B, and C (Guo et al., 2008). Twenty-one amino acids were inserted in Class A, seven amino acids in Class C, and no amino acid residues in Class B (Kotak et al., 2007; Scharf et al., 2012). Hsf plays a key role in regulating plant responses to heat shock (Lin et al., 2011; Ohama et al., 2016), drought, salt, light, and cold stress (Hwang et al., 2014; Zeng et al., 2016; Wang et al., 2020) by regulating the expression of different *Hsps*. For example, three HSFs induced by heat stress were initially cloned and identified in tomato (*Solanum lycopersicum*) (Scharf et al., 1990). Although the role of Hsps and Hsfs in stress responses is well established, the regulatory network involved in stress tolerance and the many functions of Hsps need to be further studied.

*Brachypodium distachyon* (2n = 10) is an annual temperate grass that has been widely accepted as a model plant. Its genome is completely sequenced in order to study characteristics that are unique to cereals and grasses (International Brachypodium, 2010; Catalan et al., 2014). These characteristics include small stature, rapid life cycle, genetic tractability, self-fertility, and small diploid genome size (Garvin et al., 2008; Opanowicz et al., 2008). To obtain more detailed information, we performed correlation network and functional analysis of its Hsfs and sHsps. First, we identified and surveyed phylogenetic relationships of Hsfs and sHsps*.* Then, we investigated their evolutionary relationship in terms of 3D structure, chromosomal localization, gene duplication, and cis-elements. Next, the expression patterns of *Hsfs* and *sHsps* in different tissues and several abiotic stresses were evaluated, and we established the signaling regulatory network based on the expression patterns following different stress treatments. Finally, we measured the seed germination rate and trypan blue staining of five overexpressed BdsHsps in *Arabidopsis thaliana* after heat treatment.

## Materials and Methods

### Sequence retrieval

The 23 Hsp20s and 21 Hsfs in *A. thaliana* (Scharf, Siddique & Vierling, 2001; Sarkar, Kim & Grover, 2009) and the 19 Hsp20s and 25 Hsfs in *Oryza sativa* (Guo et al., 2008; Sarkar, Kim & Grover, 2009) were obtained from the Arabidopsis Information Resources database (TAIR: http://www.arabidopsis.org/) (Lamesch et al., 2012) and the TIGR Rice Genome Annotation Resources database (http://rice.plantbiology.msu.edu/) (Ouyang et al., 2007), respectively. These sequences were used to search for the Hsp20s and Hsfs in *B. distachyon* in the publicly available PLAZA database (http://bioinformatics.psb.ugent.be/plaza/) Bel et al., 2018). Collected sequences were scanned using InterPro software and were only considered if they possessed Hsp20 or Hsf consensus sequences, as confirmed by a previous study (Mitchell et al., 2019). Any redundant or alternative splice variants were eliminated. Ultimately, we collected 18 Hsp20s and 24 Hsfs belonging to *B. distachyon*. Additionally, the molecular weights (Mw) and isoelectric points (pI) of Hsps and Hsfs were obtained from the ExPASy’s pl/Mw calculation tool (https://web.expasy.org/compute_pi/). Subcellular localization predictions for each protein were performed using the CELLO server (http://cello.life.nctu.edu.tw/).

### Gene structure, multiple sequence alignment, protein motif, and 3D structure analyses

The exon/intron organization of candidate Hsp20s and Hsfs were surveyed using Gene Structure Display Server (GSDS) software (http://gsds.gao-lab.org/; Hu et al., 2015). The sequence alignments were carried out using Clustal Omega with default parameters (http://www.ebi.ac.uk/Tools/msa/clustalo/). The Hsp20 and Hsf domains and motifs were identified using MEME (http://meme-suite.org/tools/meme; Bailey et al., 2015). Visualization of the protein-conserved domain of these genes was drawn using the WebLogo3 application (http://weblogo.threeplusone.com/). The 3D structure of the BdHsp20s and BdHsfs was modeled using PHYRE2 with default parameters (highest percent identity, most sequence coverage) (http://www.sbg.bio.ic.ac.uk/phyre2/html/page.cgi?id=index).

### Chromosomal locations, duplication, synteny, and phylogenetic analyses of *BdHsp20s* and *BdHsfs*

We collected the chromosome location information of BdHsp20 and BdHsf genes from the PLAZA database and visualized them using the CorelDRAW X3 program. Synteny information of duplicate genes was based on the PlantDGD database (http://pdgd.njau.edu.cn:8080/; Qiao et al., 2019). Phylogenetic trees based on the alignment of Hsp20 and Hsf full-length nucleotide or amino acid sequences were constructed using the maximum likelihood (ML) method with the Jones-Taylor-Thornton (JTT) model, 1000 bootstrap values, and partial deletion in MEGA 5.0 software for *O. sativa*, *A. thaliana,* and *B. distachyon*, respectively (Tamura et al., 2011).

### Cis-element analyses of *BdHsp20s* genes

The upstream 2 kb sequences of the identified *BdHsp20* genes were downloaded from the PLAZE database. The extracted sequences were submitted to the PlantCARE website (http://bioinformatics.psb.ugent.be/webtools/plantcare/html/; Lescot et al., 2002) for cis-elements analysis. The diagram of the cis-elements of *BdHsp20* genes was displayed using CorelDraw software.

### Plant growth, treatment, and expression analysis

Bd21 seeds were sown in 1/2 Murashige and Skoog (MS) medium in the dark for 4 d at 25 °C, transferred to a soil mix, and grown in a greenhouse with a cycle of 21 °C for 14 h of light and (>3000 lux)/18 °C for 10 h of darkness. Root, sheet, leaf, and caryopsis were collected from 2-week-old seedlings for tissue-specific expression analysis. For heat analysis, 2-week-old *B. distachyon* Bd21 plants were treated in liquid MS medium under 40 °C for 1 h, 3 h, 6 h, 12 h, and 24 h. For dark and UV analysis, 2-week-old *B. distachyon* Bd21 plants were treated in MS liquid medium for 1 h, 3 h, 6 h, 12 h, and 24 h. For abscisic acid (ABA) analysis, 2-week-old *B. distachyon* Bd21 plants were treated in MS liquid medium containing 100 µM ABA for 1 h, 3 h, 6 h, 12 h, and 24 h. Plants were harvested at their corresponding time points after treatment for further analyses. All samples were flash-frozen in liquid nitrogen and used for RNA extraction.

We extracted the total RNA from each sample using Trizol reagent, and reverse-transcribed 1–2 ug into cDNA using PrimeScript RT Master Mix Perfect Real-Time (TaKaRa, Kyoto, Japan) according to the manufacturer’s instructions. The total RNA was detected for quality using Nanodrop1000 and its integrity was calculated by electrophoresis in 1.5% (w/v) agarose gel. The real-time quantitative polymerase chain reaction (RT-qPCR) was executed in 10 µl reactions with 5–50 ng of first-stand cDNA products (4 µl), 5 pmol of each primer (0.4 µl), 5 µl SYBR green master mix (2X), and 0.2 µl ROX as a passive reference standard to normalize the SYBR fluorescent signal. The RT-qPCR thermal profile was as follows: initial activation at 95 °C for 5 min followed by 45 cycles of 95 °C for 30 s and 60 °C for 30 s. Afterward, PCR product specificity was monitored using a melting curve analysis (61–95 °C with fluorescence read every 0.5 °C). The *B. distachyon actin* (*Bradi2g24070*) gene was used as an internal reference for all RT-qPCR analyses. Each experiment was implemented with three independent biological replicates. The relative *BdHsp* and *BdHsf* gene expression was calculated using the 2^−ΔΔCt^ method. The up-regulated genes were defined as a fold-change greater than 2 with a *p* value of < 0.05, and the down-regulated genes were less than 0.5 with a *p* value of < 0.05. The primer-sets are listed in Table S1.

### Regulatory network and pattern analyses of *BdHsp20* and *BdHsf* genes

The length of the mRNA untranslated region (5′UTR and 3′UTR) can significantly affect mRNA stability and protein translation efficiency (Srivastava et al., 2018). To assess the relationship of 3′UTR length and the expression levels of the *BdHsp20* gene under heat stress, its correlation coefficient was investigated and the results were displayed in a linear graph. Additionally, the *BdHsp20* and *BdHsf* expression data were detected using RT-qPCR under heat stress and were clustered together to form an integrated expression profile. *BdHsf* and *BdHsp20* genes with correlation coefficients greater than 0.7 or less than −0.7 were screened as a set of their expression regulatory, which we submitted into Cytoscape software (Shannon et al., 2003) to construct their correlation regulatory network.

### Binary vector construction, Arabidopsis transformation, heat stress treatment, and trypan blue staining of the transgenic plants

We amplified full-length cDNAs encoding *BdHsp20s* (including BdHsp16.9-CI, BdHsp17.2A-CI, BdHsp17.2B-CI, BdHsp18-CII, and BdHsp16.4-CI; Table S2) using a pair of specific primers (Table S3) from Bd21 plants, cloned them into pCAMBIA1300 vector at BamHI/KpnI sites, and yielded corresponding plasmid 35S:BdHsp20s that were used for *Agrobacterium*-mediated Arabidopsis transformation. Positive transgenic plants were screened and planted on 1/2 MS medium containing 25 µg/L hygromycin for one week. The presence of the transgenic insertion was confirmed by PCR in the transgenic plants. Homozygous T3 transgenic plants were subjected to 50 °C for 1 h during the seed germination experiment. More than 100 independent plant seeds were picked for the experiment, and Arabidopsis wild-type (WT) seed was used as the control. Each treatment was conducted in triplicate independently using three biological replicates. After two weeks, the morphological observation was recorded, and the germination rate was counted.

Dead cells were determined by trypan blue staining (Cui et al., 2018; Luan et al., 2018) using the homozygous T3 transgenic plants. The T3 generation transgenic and WT seeds were treated at 50 °C for 1 h and then placed in normal growth conditions. The trypan blue staining solution contained LPTB: 25% w/v lactic acid, 2.5 mg/ml trypan blue, 25% glycerin, and 23% phenol water. The seedlings were placed in a vacuum machine and extracted for 10 min until the dye was completely injected into the plants. Afterwards, we added an appropriate amount of Chloral hydrate solution to the hood to repeat decolorization. After complete decolorization, equilibration for 2 h using 70% glycerol, and observation and photographing with a microscope, the dead blue cells were measured. The greater the number of inactive cells or cells with incomplete membranes, the deeper the trypan blue staining.

## Results

### Identification and annotation of the *Hsp20* and *Hsf* genes in *B. distachyon*

The identification and evolution of *Hsf* genes has been explored in *B. distachyon* (Table 1; Yang et al., 2014), but the relationship between *Hsp20* and *Hsf* genes needs further investigation. To identify the *Hsp20* genes, we used their sequences from *A. thaliana* and *O. sativa* as queries for BLASTP analyses in the *B. distachyon* genome database. Ultimately, a total of 18 non-redundant *Hsp20* and 24 *Hsf* sequences were retrieved (Table 1). Moreover, the genes of *Hsp20* and *Hsf* in *B. distachyon* followed the same nomenclature rule as those of *A. thaliana* and *O. sativa* based on their sequence similarities and subcellular localization, particularly Bd*Hsp20s*. For example, *Bradi1g44230* was designated as *BdHsp15.8-PX* according to its subcellular peroxisomal (PX) localization and Mw of 15.8 kD (Table 1). The results showed that most of the BdHsps (11) were located at the cytoplasm (C), except for three BdHsps in the endoplasmic reticulum, one in the PX, one in the chloroplast (CP), and two in the mitochondria (M) (Table 1). Additionally, the protein size of all *BdHsp20* proteins varied from 144aa (BdHsp15.8-PX) to 364aa (BdHsp40.0-M), while BdHsf proteins ranged from 248aa (BdHsfC1b) to 649aa (BdHsfA6a) (Table 1). Furthermore, *BdHsp20s* possessed one intron or were intronless, while all *BdHsfs,* except *BdHsfA6a* (4) and *BdHsfA1a* (2), contained one intron (Table 1). The pI of BdHsp20 proteins ranged from 4.78 (BdHsp18.7-CI) to 8.86 (BdHsp40.0-M) and the acidic proteins dominated, while the BdHsf proteins varied from 4.69 (BdHsfA2a) to 9.82 (BdHsfB1a) with the acidic proteins also occupying the main points (Table 1).

**Table 1:** Characteristics of *BdHsp20* and *BdHsf* gene families.

Gene Name	Locus ID	Orientation	ORF	No. of a.a	No. of introns	5′–3′Coordinate	pI	Mw(kD)
BdHsp16.8-CIV	Bradi1g26190	–	459	152	1	21279133–21279701	6.06	16.8
BdHsp15.8-PX	Bradi1g44230	+	435	144	0	42485161–42485595	6.92	15.8
BdHsp17.3-CI	Bradi1g53850	+	471	156	0	52437678–52438148	5.79	17.3
BdHsp16.9-CI	Bradi1g67040	+	456	151	0	65991938–65992393	5.98	16.9
BdHsp17.6-CI	Bradi1g67080	+	477	158	1	66023992–66024468	5.98	17.6
BdHsp18.7-CI	Bradi2g02350	+	537	178	1	1609756–1610292	4.78	18.7
BdHsp17.2A-CI	Bradi2g02400	–	465	154	0	1631165–1631629	6.76	17.2
BdHsp17.2B-CI	Bradi2g02410	+	462	153	0	1632105–1632566	6.19	17.2
BdHsp18.0-CII	Bradi2g05374	–	498	165	0	3913040–3913537	5.96	18
BdHsp16.4-CI	Bradi2g12990	+	444	147	0	11414902–11415345	6.19	16.4
BdHsp20.5-ER	Bradi3g02710	–	585	341	0	1675529–1676113	7.94	20.5
BdHsp22.0-ER	Bradi4g21070	+	615	204	0	24169313–24169927	6.25	22
BdHsp24.2-ER	Bradi5g11110	–	657	218	0	14808972–14809628	5.52	24.2
BdHsp18.3-CIII	Bradi3g60100	+	510	169	1	58879440–58880020	6.59	18.3
BdHsp26.4-CP	Bradi1g68440	+	720	239	1	67159967–67160773	6.77	26.4
BdHsp23.2-M	Bradi3g58590	–	642	213	1	57826850–57827614	7.9	23.2
BdHsp21.0-CV	Bradi2g20767	–	570	189	1	18240585–18242132	4.84	21
BdHsp40.0-M	Bradi3g07421	–	1095	364	1	57826850–57827614	8.86	40
BdHsfA1a	Bradi1g01130	+	1866	622	2	758757–763160	4.92	67.716
BdHsfA2a	Bradi1g37720	–	1047	349	1	33623352–33624526	4.69	38.014
BdHsfA2b	Bradi2g41530	+	1143	381	1	41768002–41770456	5.94	42.777
BdHsfA3a	Bradi3g44700	–	1536	512	1	46678418–46681158	5.52	56.011
BdHsfA4a	Bradi2g49860	+	1317	439	1	49859988–49861908	5.39	48.86
BdHsfA5a	Bradi2g18980	–	1095	365	1	16735799–16737098	5.38	40.652
BdHsfA5b	Bradi3g43710	+	1407	469	1	45299059–45301814	4.93	51.677
BdHsfA6a	Bradi1g08891	+	1947	649	4	6281780–6283033	6.43	70.343
BdHsfA6b	Bradi1g74350	–	1020	340	1	71592465–71594730	5.25	38.841
BdHsfA6c	Bradi3g26920	–	1071	357	1	27700103–27702438	5.16	40.236
BdHsfA7a	Bradi1g05550	–	1044	348	1	3777047–3779557	5.5	39.021
BdHsfA7b	Bradi1g55630	–	1374	458	1	54141088–54143900	4.88	50.304
BdHsfA8a	Bradi1g69407	–	1182	394	1	67838063–67840368	4.83	43.389
BdHsfB1a	Bradi4g32130	+	909	303	1	37847110–37850047	9.82	32.79
BdHsfB2a	Bradi3g42130	–	1038	346	1	43857313–43858451	8.1	36.641
BdHsfB2b	Bradi4g35780	–	1200	400	1	41115331–41116660	5.05	41.873
BdHsfB3a	Bradi5g18680	+	924	308	1	21774260–21775269	5.29	32.975
BdHsfB4a	Bradi1g19900	–	942	314	1	15943892–15946059	6.68	34.736
BdHsfB4b	Bradi1g61620	+	840	280	1	60967400–60969034	6.15	31.752
BdHsfB4c	Bradi4g32050	–	1215	405	1	37779303–37780616	8.97	42.982
BdHsfC1a	Bradi2g44050	+	1008	336	1	44543658–44544857	5.23	36.787
BdHsfC1b	Bradi2g48990	+	744	248	1	49157232–49158080	9.07	26.933
BdHsfC2a	Bradi1g38140	+	759	253	1	34296765–34297648	6.11	26.937
BdHsfC2b	Bradi3g08870	+	945	315	1	6966950–6968174	6	33.332

**Notes.**

C, Cytoplasmic; CP, Chloroplast; ER, endoplasmic reticulum; M, Mitochondrial and PX, peroxisomal.

### Phylogenetic classification and gene structure of BdHsp20s and BdHsfs

**Figure 1:**
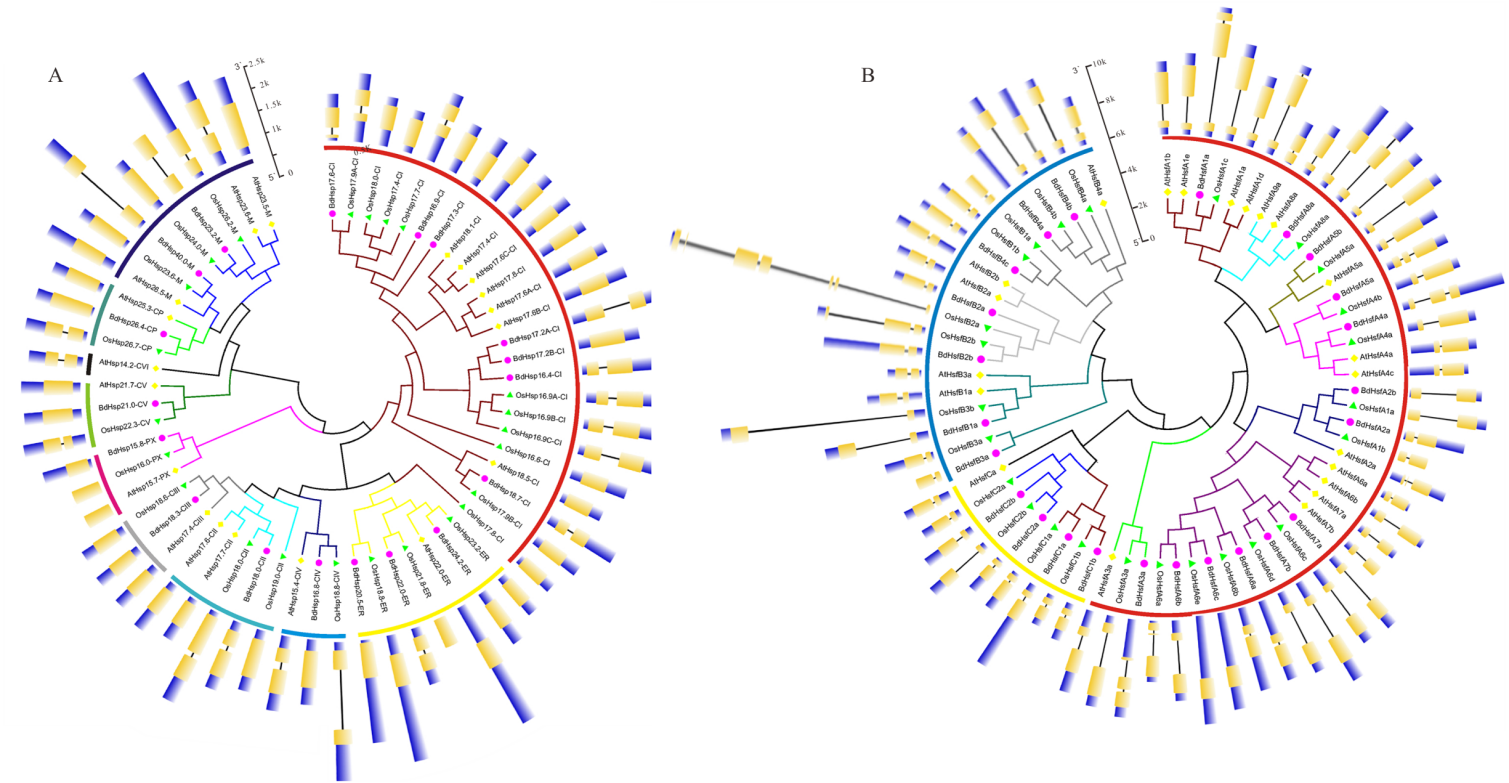
Phylogenetic relationships and gene structure analysis of Hsf (A) and Hsp20 (B) genes from *O. sativa*, *A. thaliana*, and *B. distachyon*, respectively.

To evaluate the evolutionary relationship between BdHsp20s and BdHsfs, the phylogenetic tree was independently reconstructed based on the sequence alignments of *B*. *distachyon*, *A*. *thaliana,* and *O*. *sativa* full-length amino acids (Table S4) and using the ML method. According to our phylogenetic analysis and subcellular localization prediction results, the BdHsp20s could be divided into cytoplasmic (CI, CII, CIII, CIV, CV, and CVI), CP, ER, M, and PX subfamilies (Fig. 1A). Most Hsp20s belonged to the CI subfamily, suggesting that the cytoplasm may be the main functional region of the plant Hsp20s. The CVI subfamily had only one member and was derived from *A*. *thaliana*, while *B*. *distachyon* and *O*. *sativa* had no members (Fig. 1A), implying that CVI Hsp20 might only exist in dicotyledons. We also found a close relationship between the M and CP subfamilies (Fig. 1A), which supports the results of a previous study (Waters, Aevermann & Sanders-Reed, 2008). It was interesting that the classification of subfamilies was closely consistent with the pattern of intron-exon structure, as supported by previous research (Ouyang et al., 2009).

Likewise, phylogenetic analysis divided Hsfs into three main clades, designated as classes A, B, and C (Fig. 1B). Class A Hsfs were further divided into nine groups based on their phylogenetic relationships: subgroup A1, A2, A3, A4, A5, A6, A7, A8, and A9. Of these nine groups, most of the subgroups contained genes in three plant genomes, except for subgroup A9 which was only in *A*. *thaliana* and subgroup A2 and A7 which were absent in *O*. *sativa* (Fig. 1B). Moreover, class B was divided into four subgroups (B1, B2, B3, and B4), while class C Hsfs were separated into C1 and C2. It was notable that there was only one member in *A*. *thaliana*, while class C Hsfs were amplified in *B*. *distachyon* and *O*. *sativa* (Fig. 1B)*,* indicating that these multiple gene copies may exhibit expansion in monocots. Furthermore, we also found that the subgroup classification patterns were related to intron-exon organization, *e.g.*, class C Hsfs had fully similar intron numbers, exon lengths, and intron phases (Fig. 1B).

### Chromosomal location and gene duplication of *BdHsp20s* and *BdHsfs*

**Figure 2:**
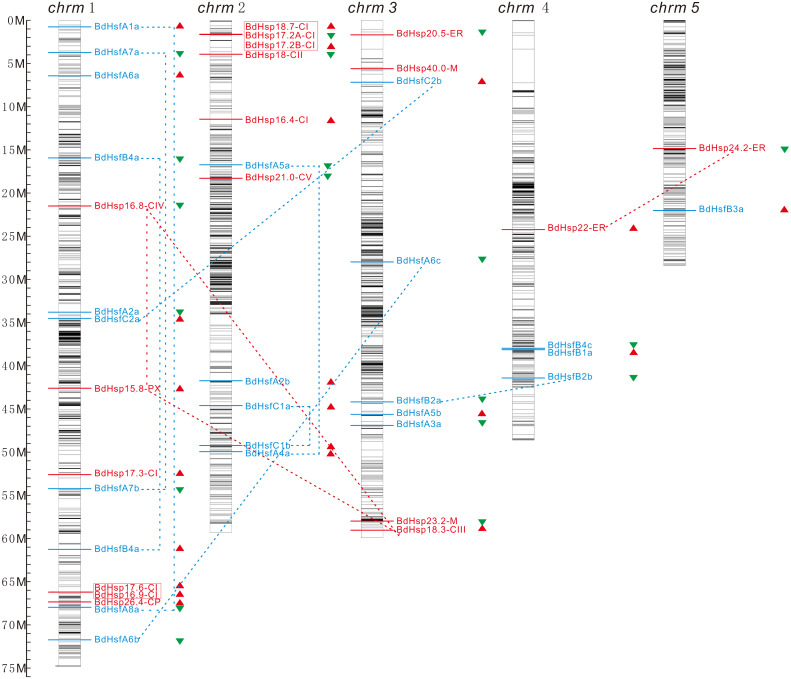
Chromosomal location and gene duplication analyses for *B. distachyon Hsp20* and *Hsf* genes. The chromosomal position of each *BdHsf* and *BdHsp20* gene was mapped according to the *B.* distachyon genome. The chromosome number is indicated at the top of each chromosome. The CpG island distribution maps shown in each chromosome depended on the CpG density in the *B. distachyon* genome. The colored lines and characters represent the subgroup to which proteins in each clade belong. The dotted line shows the gene duplication events across these *BdHsf* and *BdHsp20* genes. The scale bar is shown on the left.

To analyze the relationship between the gene duplication and genetic divergence within *BdHsp20s* or *BdHsfs*, we investigated their chromosomal locations based on the information from the PLAZA database. The physical locations and CpG island distributions of the *BdHsp20* and *BdHsf* genes on *B. distachyon* chromosomes were drawn (Fig. 2). The results showed that the 18 *BdHsp20s* and 24 *BdHsfs* were mapped on five *B. distachyon* chromosomes: 16 genes were located on chromosome 1, 11 genes on chromosome 2, nine genes on chromosome 3, four genes on chromosome 4, and only one Hsf and Hsp20 member on chromosome 5 (Fig. 2). Interestingly, all *BdHsp20* and *BdHsf* gene members were encoded at the low-density region of CpG islands on all five chromosomes, suggesting that the expression of these genes may be regulated by other genes, which was consistent with previous studies (Bird, 1986; Hahn et al., 2011).

Gene duplication events, such as tandem duplication and segmental duplication, might generate new members of a gene family because of the important role that base substitution, deletion, and insertion into different chromosomes play in the expansion of gene families (Cannon et al., 2004). Therefore, we surveyed the duplication events of *BdHsp20s* and *BdHsfs*. We found that five pairs of duplicates underwent tandem duplication and 12 pairs of gene duplicates underwent segmental duplication (Fig. 2), indicating that tandem duplication and segmental duplication might contribute to the amplification of these gene families. These results showed that the greater number of gene family members might be the result of genomic rearrangement and expansion during the evolution process.

### Protein structure and tissue-specific expression profile of *BdHsp20s* and *BdHsfs*

**Figure 3:**
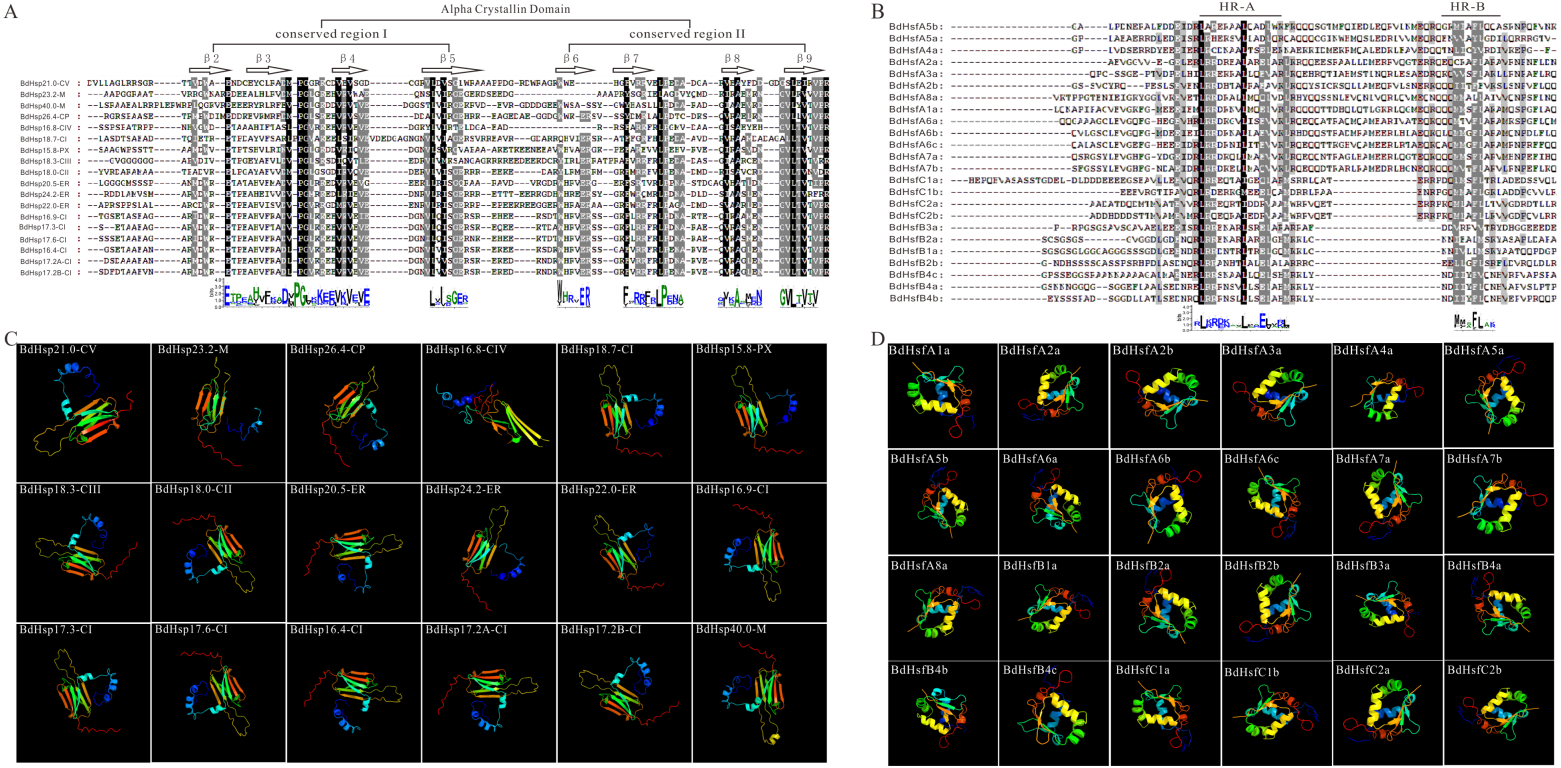
Multiple-sequence alignment analysis and three-dimensional structural model of BdHsfs and BdHsp20s. (A–B) Alignment of selected conserved domains of BdHsf and BdHsp20 amino acid sequences. (C–D) Three-dimensional structural model of BdHsf and BdHsp20 proteins.

To better understand BdHsp20 and BdHsf characteristics, we further analyzed their sequence features, specifically the ACD of BdHsp20s and the HR-A/B of BdHsfs (Fig. 3A and 3B). As expected, all BdHsp20s contained an ACD consensus sequence that was composed of two regions: CRI and CRII (Fig. 3A). CRI was formed by β2, β3, β4, and β5, while CRII was formed by β6, β7, β8, and β9. However, BdHsp16.8-CIV and BdHsp23.2-M lacked a β6 structure that played an important role in the normal formation of dimers (Siddique et al., 2008), which was also supported by the 3D structure of the two proteins (Fig. 3C), indicating that they might be replaced by another BdHsp20 member in performing biological functions. Additionally, the typical BdHsf HRA/B contained approximately 100 conserved amino acids. Using the difference of the flexible linkers between the HR-A and B regions, BdHsfs were divided into three categories: Class A (13), Class B (7), and Class C (4) (Fig. 3B). The HR-A containing the most conserved amino acids was L-K/R-R-R-D/E-X_3_-L-X_2_-E-L/V, and M-M­X-F-L-X-K/R contained the most in HR-B (Fig. 3B). Indeed, the 3D structure of the 24 BdHsfs constructed using phyre2 showed several conserved motifs, including DBD, NLS, and NES (Fig. 3D).

**Figure 4:**
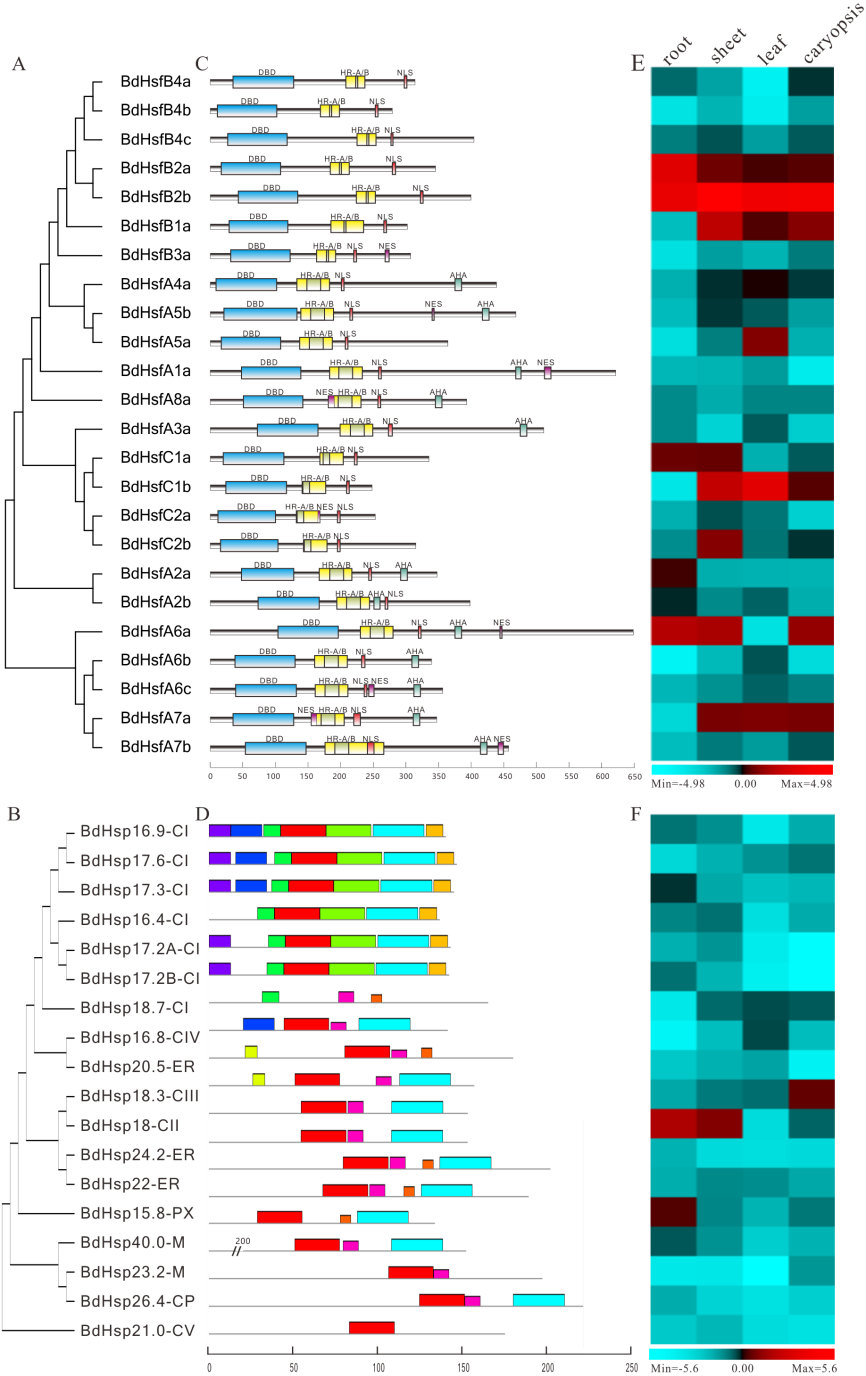
Phylogenetic relationships and protein structure analysis of BdHsf and BdHsp20 proteins with tissue-specific expression profiles. (A–B) The phylogenetic tree was constructed from the amino acid sequences using the ML program from MEGA 5, and represents relationships between BdHsf and BdHsp20 proteins. (C–D) Domain analysis of BdHsf and BdHsp20 proteins. (E–F) Expression patterns of *BdHsf* and *BdHsp20* genes in different tissues including root, sheet, leaf, and caryopsis, respectively.

Moreover, the phylogenetic tree of full-length BdHsf and BdHsp20 proteins was reconstructed using MEGA 5.0 (Fig. 4A and 4B). The clustering result was slightly different from the clustering based on the protein sequences of *B. distachyon*, *A. thaliana*, and *O. sativa*. Remarkably, although the gene coding regions had different lengths, the BdHsf domains were highly identical. All the BdHsf members contained DBD, HR-A/B, and NLS domains, while class B BdHsfB3a shared a NES motif and class A BdHsfs also contained an AHA domain that was different from BdHsfA5a (Fig. 4C). In addition, seven class A BdHsf members also had a NES domain similar to BdHsfA6a’s (Fig. 4C). A schematic representing the structure of all BdHsp20 members was drawn using the analysis results from the MEME server (Fig. 4D). We found that all BdHsp20s contained an ACD conserved domain, which was composed of a CRI region (β2, β3, β4, and β5) formed by motif 1 and motif 4, and a CRII region (β6, β7, β8, and β9) formed by motif 2 and motif 3 (Fig. S1). Interestingly, the clustered BdHsp20 pairs localizing the same cellular compartments also exhibited highly similar motif organization, such as BdHsp18.3-CIII/BdHsp18-CII and BdHsp24.2-ER/BdHsp22-ER (Fig. 4D), indicating that the protein architecture was helpful in the distribution and aggregation of the protein.

We also investigated the expression patterns of *BdHsf* and *BdHsp20* genes in four tissues or organs (root, sheet, leaf, and caryopsis) using the RT-qPCR method. We found that *BdHsfB2a* and *BdHsfB2b* were highly expressed in four tissues; *BdHsfB1a*, *BdHsfC1b,* and *BdHsfA7a* had a relatively high expression level in all tissues except for root; and *BdHsfA6a* was highly expressed in root, sheet, and caryopsis (Fig. 4E). Other *BdHsf* genes displayed a relatively low expression level in all tissues (Fig. 4E). Additionally, most *BdHsp20* gene members also showed low expression in all tissues, except for a few genes with moderate or high expression levels (Fig. 4F). For example, BdHsp18-CII showed high expression in root and sheet, *BdHsp18.3-CIII* showed high expression in caryopsis, and *BdHsp17.3-CI* and *BdHsp15.8-PX* were moderately expressed in the root (Fig. 4F). These results suggested that *BdHsf* and *BdHsp20* genes may not be expressed during normal growth and development, but are rapidly and highly expressed in response to stresses.

### Cis-elements and expression analysis of *BdHsp20s* and *BdHsfs* under multiple abiotic stresses and ABA treatment

**Figure 5:**
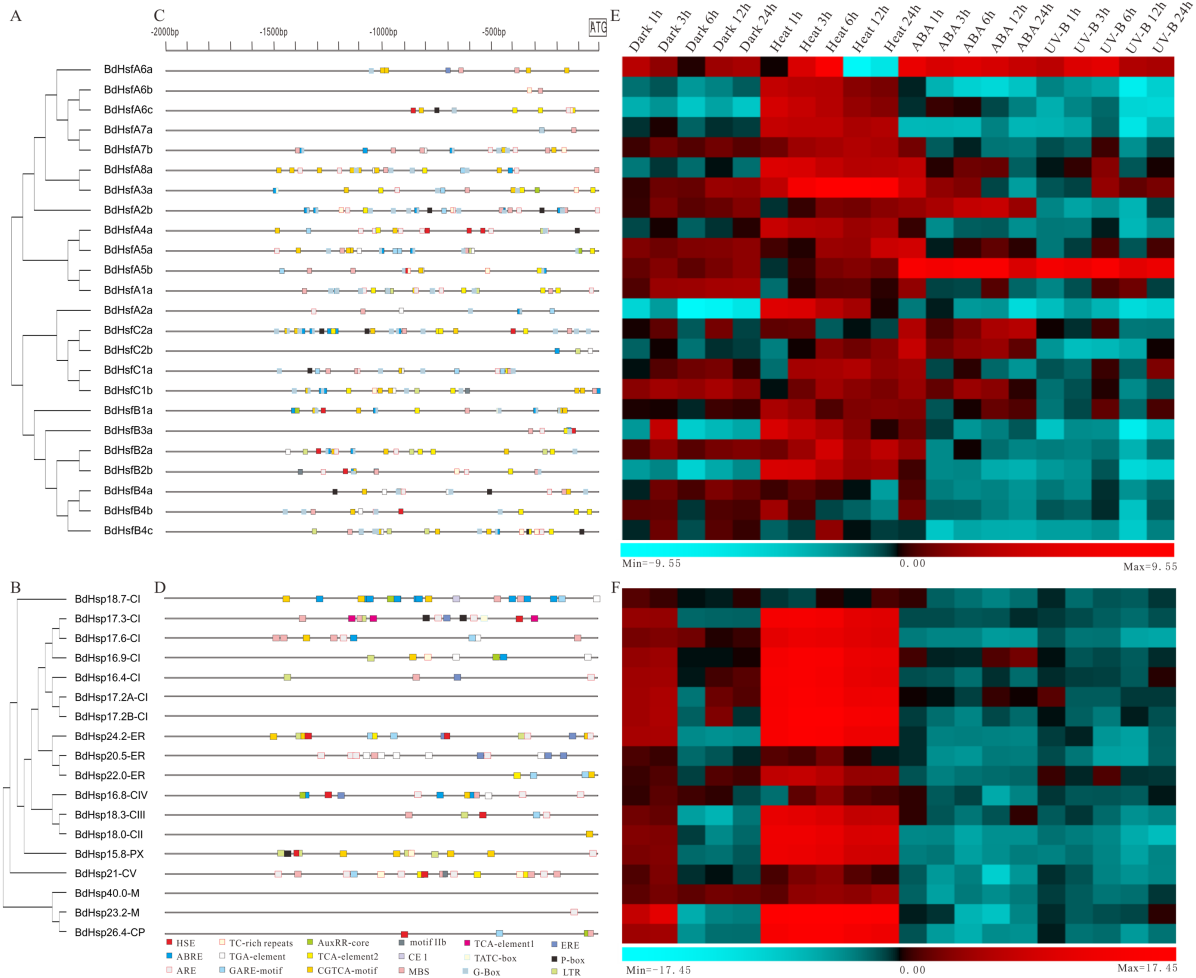
Phylogenetic relationships and cis-element analysis with expression profiles of *BdHsf* and *BdHsp20* genes under different abiotic stresses. (A–B) The phylogenetic tree was constructed from the CDS sequences using the ML program from MEGA 5, and represents relationships between BdHsf and BdHsp20 genes. (C–D) Promoter analysis of *BdHsf* and *BdHsp20* genes. All promoter sequences (−2000 bp) were analyzed. The scale bar at the top indicates the length of the promoter sequence. (E–F) Expression patterns of *BdHsf* and *BdHsp20* genes under different abiotic stresses including dark, heat, ABA, and UV-B treatments.

To explore the function of *BdHsp20s’* and *BdHsfs’* response to abiotic stresses and hormones, the Bd21 seedling expression profiles were examined using RT-qPCR under dark, heat, UV-B, and ABA treatments. We first investigated the cis-elements in the -2 kb promoter region of the *BdHsf* and *BdHsp20* genes, except for *BdHsp17.2A-CI*, *BdHsp17.2B-CI,* and *BdHsp40.0-M* on account of their lack of a promoter region. The clustering result was obtained based on the phylogenetic tree of the full-length CDS sequence (Fig. 5A and 5B). We found that *BdHsp20s* and *BdHsfs* contained various cis-elements involved with defense and hormone and stress responsiveness (Fig. 5C and 5D). For instance, almost all *BdHsf* genes had G-Box and MBS elements, while most genes lacked motif IIb, ERE, and TCA-element 2, even all TATC-box and CE I (Table S5), suggesting that *BdHsf* genes may be mainly induced by abiotic stresses, not hormones. Likewise, only one *BdHsp20* gene member contained one cis-element including TATC-box, TCA-element 2, CE I, and motif IIb which associated with hormone response, while most *BdHsp20* gene members contained ARE and MBS elements (Table S5). Most importantly, seven *BdHsp20* genes (*BdHsp16.8-CIV*, *BdHsp15.8-PX*, *BdHsp17.3-CI*, *BdHsp24.2-ER*, *BdHsp18.3-CIII*, *BdHsp26.4-CP,* and *BdHsp21.0-CV*) contained HSE elements (Table S5).

We then investigated the transcript levels of *BdHsp20s* and *BdHsfs* under multiple abiotic stresses and ABA treatment. The results showed that most *BdHsp20s* and *BdHsfs*, such as *BdHsfA3a*, were strongly induced by heat stress, were moderately regulated by dark treatment, and were almost not affected by ABA and UV-B treatments (Fig. 5E and 5F). However, there were some differences in the expression profiles of some genes. For example, the expression levels of *BdHsfA6a* and *BdHsfA5b* significantly increased after ABA and UV-B treatments, while *BdHsfA2b* increased in the ABA condition (Fig. 5E), which was consistent with their corresponding cis-element distributions. The expression levels of *BdHsfA6a* were up-regulated after heat stress, peaking at 6 h, and then recovered to relatively low levels (Fig. 5E and Table S6). Interestingly, many *BdHsp20* genes (including *BdHsp23.2-M* and *BdHsp26.4-CP)* showed growth under early dark treatment, peaking at 3 h, and then dropping to very low levels (Fig. 5F). Heat stress had no obvious influence on the expression of *BdHsp18.7-CI*, *BdHsp20.5-ER,* and *BdHsp16.8-CIV*, which may be due to their missing HSE element (Fig. 5F and Table S6). These results indicated that *BdHsp20s* and *BdHsfs* were all strongly regulated by heat stress and regulated the expressions of downstream genes.

To further survey the potential factor of gene expression, we calculated the correlation coefficient between 3′UTR length and *BdHsp20* gene expression under heat stress. The result revealed that all of the *BdHsp20* genes had a significant negative correlation with their 3′UTR length (Fig. S2). The shorter 3′UTR length, the higher expression of *BdHsp20* genes under heat stress, which may be because of the escape from the inhibition of some miRNAs (Zhang et al., 2019). However, under heat stress, the 5′UTR length had no significant effect on the expression of *BdHsp20* genes, which fully conflicted with the correlation of 3′UTR (Fig. S3). Even so, we found that the 5′UTR lengths showed a negative correlation with gene expression levels of *BdHsp20* genes under heat stress, which was in accordance with a previous study (Rao et al., 2013).

### *BdHsp20* and *BdHsf* gene regulatory network

**Figure 6:**
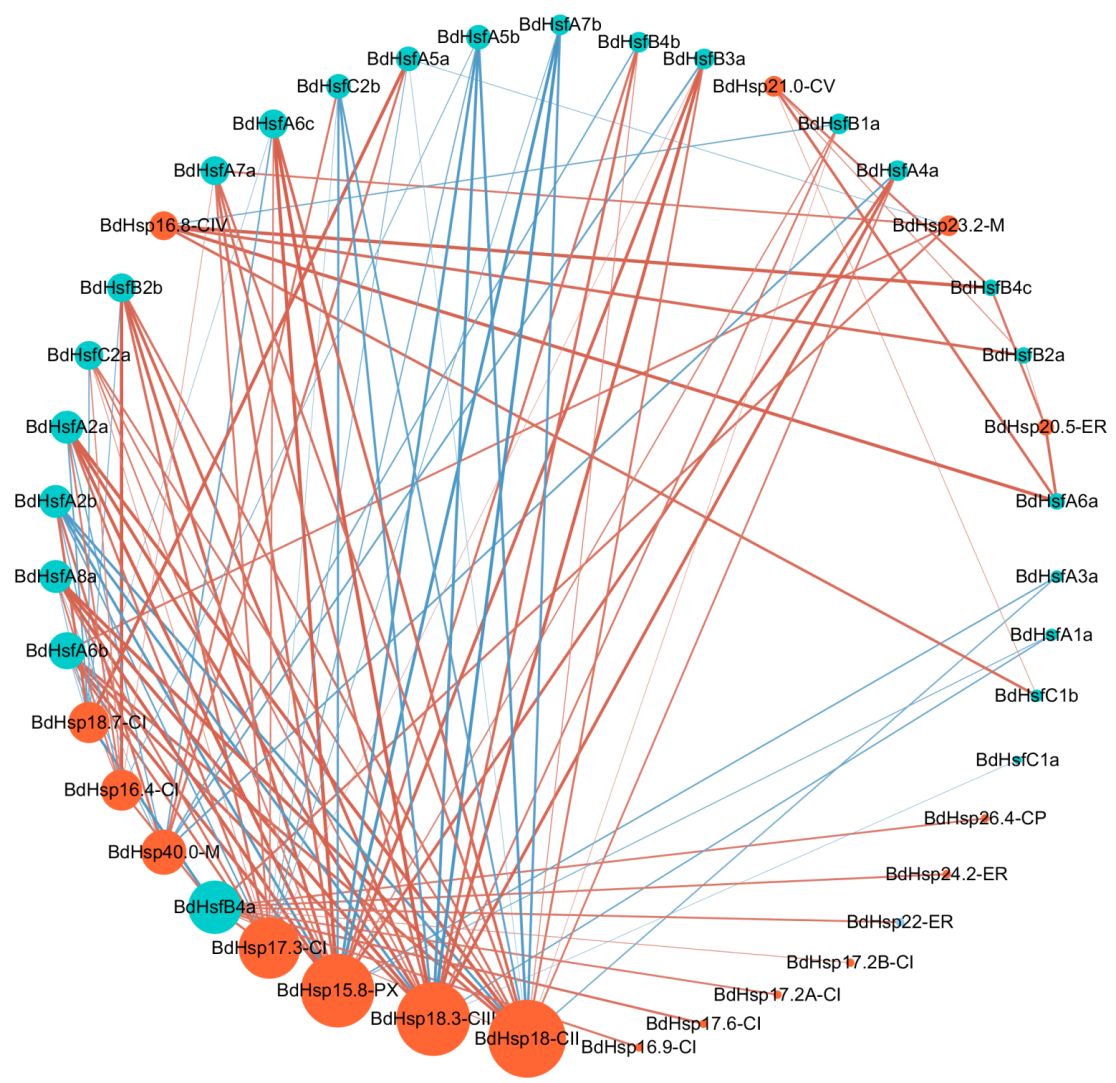
Visualization for the predicted results of the expression network between BdHsfs and BdHsp20s, constructed using the Cytoscape tool. Each node represents a protein, and each line represents an interaction. The orange and blue node color indicates the *BdHsp20* and *BdHsf* gene families, respectively. Line thickness reflects the correlation strength of the expression. Font size and circle color indicate the correlated expression number. Our results showed that there were 143 pairs of potential expression pairs. The network was generated using the Cytoscape tool.

Together, Hsfs and Hsp20s usually formed signaling modules in response to environmental stresses, especially heat shock stimuli (Lin et al., 2011). Hsp20 expression can be strongly induced by heat and other abiotic stresses, while Hsfs can initially perceive the stimuli and facilitate the expression of Hsps by binding to the HSE of the *Hsp* promoter region (Schoffl, Prandl & Reindl, 1998). To explore the potential regulatory network between *BdHsfs* and the downstream protein BdHsp20s under heat stress, we calculated the correlation coefficient between their expression results and constructed the correlation expression network (consisting of 111 nodes and 402 interactions) using Cytoscape software (Fig. 6). We selected the molecular modules if the values of their correlation coefficients were greater than 0.7. The results showed that eight *BdHsp20* genes (*BdHsp18-CII*, *BdHsp18.3-CIII*, *BdHsp15.8-PX*, *BdHsp17.3-CI*, *BdHsp40.0-M*, *BdHsp16.4-CI*, *BdHsp18.7-CI*, and *BdHsp16.8-CIV*) were regulated by almost all *BdHsf* genes (Fig. 6). For instance, the *BdHsp18-CII* gene could be regulated by 12 *BdHsf* genes, while *BdHsp15.8-PX* and *BdHsp18.3-CIII* could be regulated by 11 *BdHsf* genes (Fig. 6). Other *BdHsp20* genes might be regulated by other *BdHsf* genes under other abiotic stresses. Moreover, all *BdHsf* genes, except *BdHsfC2b,* could regulate more than one *BdHsp20* gene. For example, *BdHsfB4a* could regulate eleven *BdHsp20* genes (Fig. 6). Furthermore, it was notable that *BdHsfA5b* and *BdHsfA7b* expression showed significant negative correlation with *BdHsp20s*, suggesting that they may be transcriptional repressors. These results indicated a complex signaling regulatory network between *BdHsp20s* and *BdHsfs*, which may exhibit the same functions or interactions in response to heat stress treatment.

### *BdHsp20* gene overexpression improved plant seed germination under heat stress

**Figure 7:**
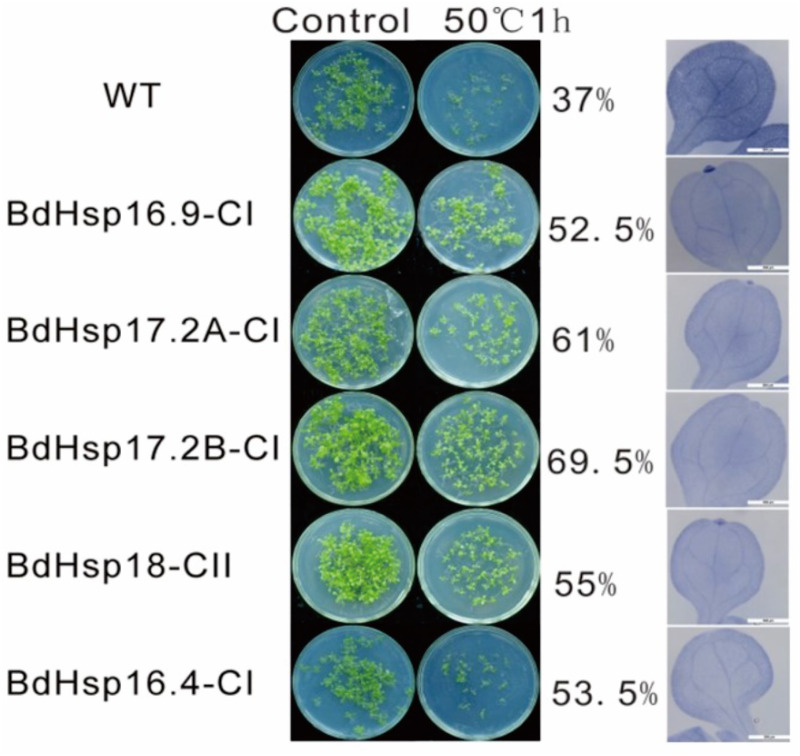
The effect of BdHsp20 overexpression on seed germination and leaf cell death after heat treatment.

To investigate the effect of high-temperature stress on *BdHsp20* seed germination, we examined the seed germination rate and trypan blue staining assays of heterologous expression in *A. thaliana* across five *BdHsp20* genes. We found that the germination rate of *BdHsp20* genes in transgenic plants was higher than in WT seedlings (Fig. 7 and Table S7). For example, the germination rate of BdHsp17.2B-CI transgenic plants was 69.5 after treatment, while WT seedlings showed a germination rate of 37 (Fig. 7 and Table S7). This finding indicated that *BdHsp20* transgenic plants had a higher survival rate under high-temperature stress. To further explore the details of cell survival under high-temperatures, the trypan blue staining of the leaf blade was executed. The results showed that the WT leaf was a deeper blue when compared to *BdHsp20* transgenic leaves under high-temperature stress (Fig. 7), indicating that there was more cell death in WT plants. Therefore, we concluded that these *BdHsp20* genes can enhance resistance to heat stress.

## Discussion

### Phylogeny, evolution, and duplication analyses of *BdsHsp20* and *BdHsf* gene families

In this study, we investigated the evolution of *BdsHsp20* and *BdHsf* gene families. We first retrieved 18 *sHsp20s* and 24 *Hsfs* in *B. distachyon* and then reconstructed their ML phylogenetic trees (Fig. 1). *B. distachyon* BdHsfs and BdsHsp20s were divided into three and ten subfamilies, respectively (Fig. 1), similar to *A. thaliana* and rice (Scharf et al., 2012). Further analysis showed that the pattern of *BdsHsp20* and *BdHsf* gene families’ intron-exon organization was closely related to their phylogenetic branch (Fig. 1). In addition, *BdHsp20s* and *BdHsfs* were all located in the low-density region of CpG islands, and were distributed tendentiously on all five chromosomes (Fig. 2), which was in accordance with previous research (Jiang et al., 2015). We also found that four pairs of duplicates showed tandem duplication in subgroup CI *BdsHsp20s* (Fig. 2), suggesting that tandem duplication contributed to the expansion of these genes, similar to the patterns of the *MKK* gene family (Jiang & Chu, 2018; Jiang, Li & Wang, 2021). Moreover, domain and 3D structure analyses showed that *BdHsp20s* and *BdHsfs* were highly conserved (Fig. 3). Most importantly, these results indicated that the *BdsHsp20* and *BdHsf* gene families were highly conserved.

### Correlation regulatory networks across *BdHsp20* and *BdHsf* genes

Numerous *Hsp* and *Hsf* genes have been reported in higher plants such as *Prunus mume* (Wan et al., 2019) and *Populus trichocarpa* (Zhang et al., 2015). It is well-known that tissue-specific expression patterns may enable Hsp and Hsf proteins to have distinct functions in different signaling pathways, although Hsp and Hsf proteins usually show low abundance under normal conditions. For example, *PtHsf-A2* showed high transcript levels in internode 9, *PtHsf-B2a* showed high transcript levels in internode 5, and *PtHsf-A6a* showed high levels in female catkins, which was in accordance with a previous study that reported that these genes are key regulators of downstream *Hsps* (Nishizawa et al., 2006). Therefore, we examined the expression profiles of different tissues in *B. distachyon*. *BdHsfB2a* was highly expressed in four tissues, and *BdHsfA6a* in root, sheet, and caryopsis (Fig. 4E), implying that they might have a unique signaling pathway. Moreover, almost all *BdHsp20s* and *BdHsfs* were significantly up-regulated under heat stress (Fig. 5E), indicating that these genes play a significant role in heat stress response. Furthermore, correlation networks between *PtHsfs* and *PtHsps* under various stresses (Zhang et al., 2015) and HSF/HSP with switchgrass Cd tolerance (Song et al., 2018) have been constructed. In this study, *BdHsfB4a*, *BdHsfA5b* and *BdHsfA7b* revealed high correlation levels with BdsHsps (Fig. 6), suggesting that these *BdHsfs* may be the key regulators for many BdsHsps. On the other hand, several *BdHsp20s*, including *BdHsp18-CII*, *BdHsp18.3-CIII,* and *BdHsp15.8-PX*, displayed co-expression with *BdHsfs* (Fig. 6), indicating that these genes might play a crucial role in the heat stress response. However, the regulatory mechanisms of herbaceous Hsf and sHsp plant growth, development, and stress responses require further study.

### Functional analysis of *BdHsp20s* in *B. distachyon* under high temperatures

Previous studies suggested that *VcHSP17.7* was associated with the chilling injury resistance of blueberry (*Vaccinium corymbosum)* fruit induced by heat shock treatment (Shi et al., 2017). *Rosa chinensis RcHsp17.8* confers stress tolerance to heat, cold, salt, drought, and osmotic and oxidative conditions (Jiang et al., 2009), while *OsHSP18.6* overexpression improved thermotolerance (Wang et al., 2015). In this study, *BdHsp17.2B-CI* expression was strongly up-regulated under heat conditions (Fig. 5), whereas overexpression in *A. thaliana* plants enhanced their tolerance to heat stress by decreasing cell death (Fig. 7), indicating that *BdHsp17.2B-CI* conferred resistance to heat stress. Similarly, *AtHSP17.4* and *CsHSP17.2* were also more actively expressed in transgenic overexpressed plants under heat shock (Wahid et al., 2007; Wang et al., 2017). The rest of the four overexpressed *BdHsp20s* in *A. thaliana* also showed improved tolerance to heat stress (Fig. 7), which supported previous transgenic research (Guo et al., 2020). The heterologous expression of LimHSP16.45 in Arabidopsis enhanced their tolerance to various stresses by forming heat shock particles (HSGs) (Mu et al., 2013). Although it is well-known that *HSP20* genes can be induced by heat stress, some heat-regulated genes were almost expressed or downregulated under UA-B and/or dark conditions (Fig. 5F), suggesting that *HSP20* genes may have a negative or only slight effect under UA-B and/or dark stress. These results suggest that the signaling pathways may be different in a plant’s response to heat and light stress. Collectively, these results provide a basis for further functional research on the roles of these families in herbaceous plant stress responses.

## Conclusion

In this study, we retrieved 24 *Hsf* and 18 *sHsp* genes in *B. distachyon* using bioinformatics approaches. Based on the phylogenetic tree, subcellular localization, and domain analyses, the *BdHsfs* were categorized into three classes, and the BdHsp20s were divided into ten subfamilies. Notably, we found that 3′UTR length might negatively affect *BdHsp20* gene expression. Expression analysis showed that most *BdHsp20s* and *BdHsfs* were strongly and rapidly regulated by heat stimuli, moderately induced by dark treatment, and almost not affected at all by ABA and UV-B treatments. More importantly, the correlation regulatory network between *BdHsp20s* and *BdHsfs* under heat stress treatments showed a complex signaling regulatory network. Additionally, seed germination and trypan blue staining analyses revealed that *BdHsp20s* enhanced high-temperature tolerance. These results provide useful information for future research on the regulatory networks of *BdHsp20s’* and *BdHsfs’* response to environmental stresses, and improved our understanding of the functions of *Hsf* and *sHsp* genes in herbaceous plants.

##  Supplemental Information

10.7717/peerj.12267/supp-1 Supplemental Information 1 Details of BdHsp20s sequence logo of motifs Logos were generated using the Weblogo3 application (http://weblogo.threeplusone.com/).

10.7717/peerj.12267/supp-2 Supplemental Information 2 Relationships between 3′UTR length and expression of *BdHsp20* genes under heat stress

10.7717/peerj.12267/supp-3 Supplemental Information 3 Relationships between 5′UTR length and expression of *BdHsp20* genes under heat stress

10.7717/peerj.12267/supp-4 Supplemental Information 4 A list of RT-qPCR primers of *Hsp20* and *Hsf* genes in *B. distachyon*

10.7717/peerj.12267/supp-5 Supplemental Information 5 Complete DNA sequences of five Hsp20s constructing overexpression vector and the associated maps used in this work

10.7717/peerj.12267/supp-6 Supplemental Information 6 Gene-specific primers used in overexpression plasmid construction

10.7717/peerj.12267/supp-7 Supplemental Information 7 List of *Hsp20* and *Hsf* genes in *A. thaliana* and *O. sativa.*

10.7717/peerj.12267/supp-8 Supplemental Information 8 Details of cis-elements in BdHsp20 and BdHsf promoter regions

10.7717/peerj.12267/supp-9 Supplemental Information 9 Expression data of *BdHsp20* and *BdHsf* genes under various treatments

10.7717/peerj.12267/supp-10 Supplemental Information 10 Comparsion of germination rate between transgenic and WT seeds

## Additional Information and Declarations

### Data Availability

The following information was supplied regarding data availability: The data of RT-qPCR experiments are available in the Supplemental Table.
