# Supplementary figures and images for: Identification, expression, and functional analysis of Hsf and Hsp20 gene families in Brachypodium distachyon under heat stress
Source: PeerJ. 2021 Oct 1;9:e12267. doi: 10.7717/peerj.12267 (PMC8489411; doi:10.7717/peerj.12267)

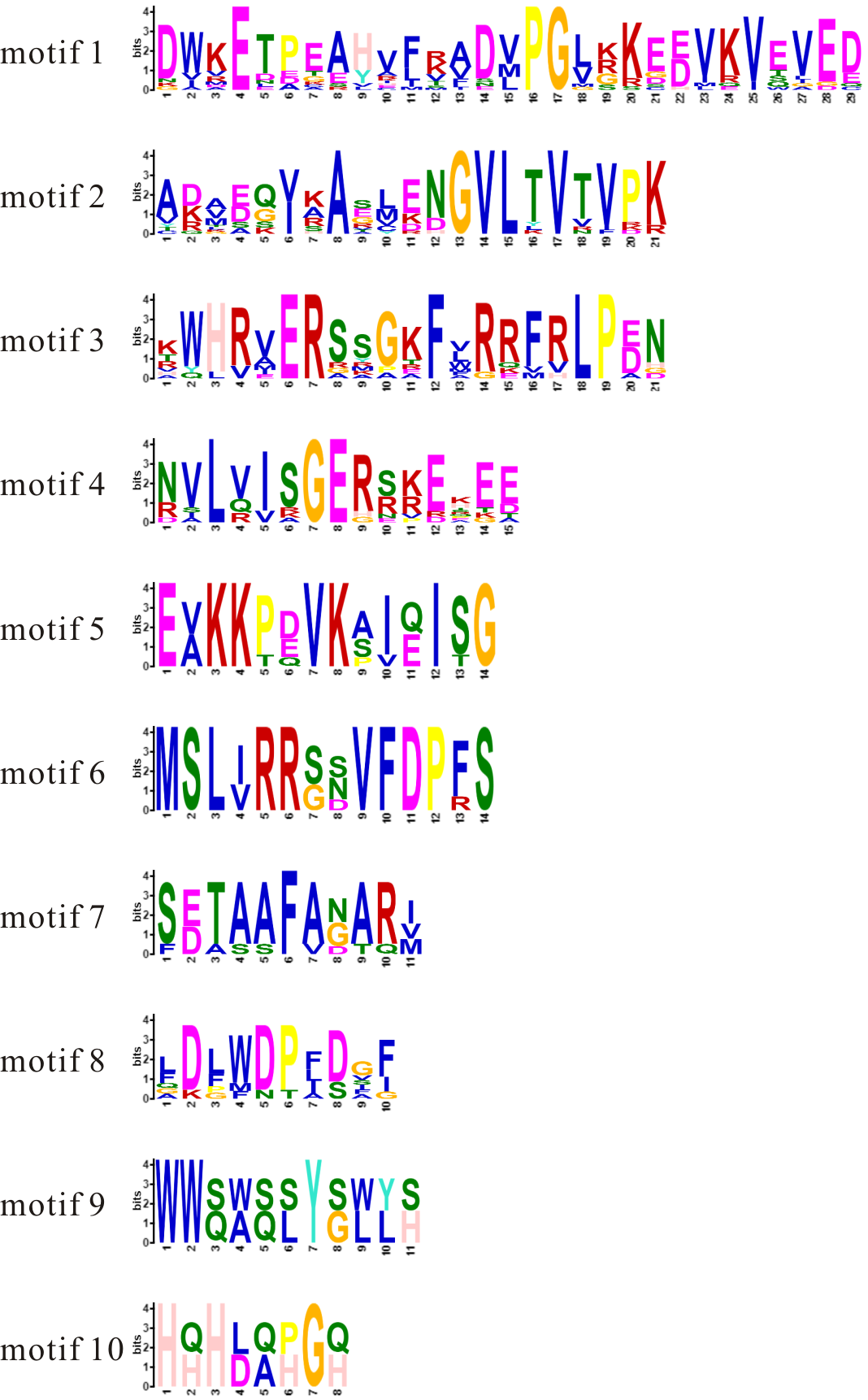

Supplement: Supplemental Information 1 — Logos were generated using the Weblogo3 application (http://weblogo.threeplusone.com/). [file peerj-09-12267-s001.tif]

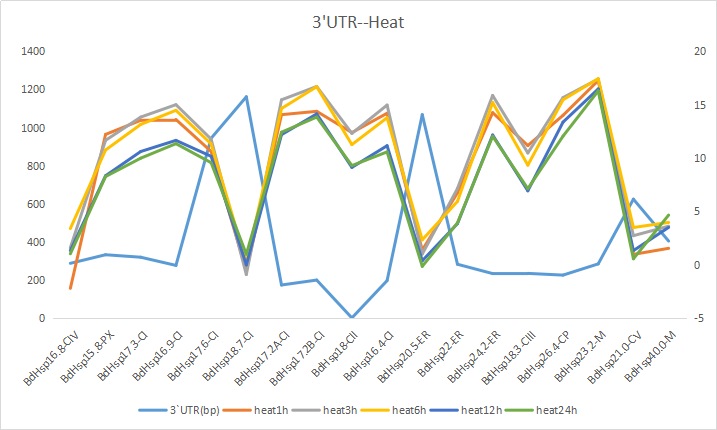

Supplement: Supplemental Information 2 [file peerj-09-12267-s002.png]

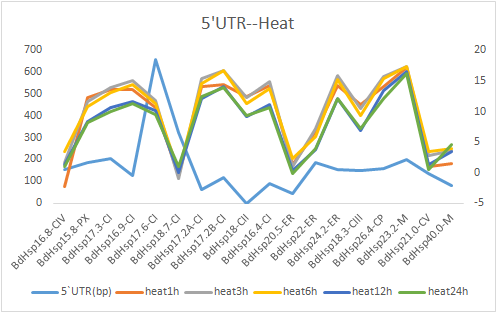

Supplement: Supplemental Information 3 [file peerj-09-12267-s003.png]
