# Supplementary material for: Identification, expression, and functional analysis of Hsf and Hsp20 gene families in Brachypodium distachyon under heat stress
Source: PeerJ. 2021 Oct 1;9:e12267. doi: 10.7717/peerj.12267 (PMC8489411; doi:10.7717/peerj.12267)
Supplement: Supplemental Information 4 [file peerj-09-12267-s004.docx]

Table S1. **The list of RT-qPCR primers of *Hsp20* and *Hsf* genes in *B. distachyon* were shown.**

| Gene Name | Forward Primer | Reward Primer |
| --- | --- | --- |
| BdHsp16.8-CIV | AGTGGCAGCTCTACTCCCTC | GGTATTTCCCGTCCTCCACC |
| BdHsp15.8-PX | TGGTACTGGAAAGCTCGGTG | TATTCCATGCCGCTTCGCTC |
| BdHsp17.3-CI | TCCATCCAGATCTCCGGCTA | TGACTCGACGGCATCACAAT |
| BdHsp16.9-CI | CACGAGGAGAAGTCCGACAC | CCGGTGATCTGAACTGGCTT |
| BdHsp17.6-CI | GCACGTGTTCAAGGCGGAT | CTTCTCCTCGTGCTCCTTGTTG |
| BdHsp18.7-CI | GTAGCCGGGCCACTTTCTT | CAACGGCACCCACTCTGG |
| BdHsp17.2A-CI | CTGCTTGTGTGAACGGTGTG | TTCAGGCGAACAGAGGACAG |
| BdHsp17.2B-CI | GACTGGAAGGAGACTCCCGA | TCTGGCTTCTTGACCTCAGAC |
| BdHsp18.0-CII | AGAACGCCGACATGGACAAG | GACCTGGACGTTGATGGTCTT |
| BdHsp16.4-CI | CGACTTCTGGGACCCTCTG | TGACCTCCTCTTTCTTCACGC |
| BdHsp20.5-ER | CGTCCTCACCGTTACCATCC | ACTGCCCCATGTACGTCTTC |
| BdHsp22.0-ER | TGTTTACGGCGGCTGTTCT | TCTCCTTCCAGTCGCACCTC |
| BdHsp24.2-ER | GTGTGCTTACTGTGCGGTTC | CGCTCTTCACCGCCAATACT |
| BdHsp18.3-CIII | TGGACACGGACATGAGCAAG | AGAGTCACCTGGATGTCGGA |
| BdHsp26.4-CP | AGCGTGAGCTCCTACGACAT | GTGCTCCGTCTCTGTCTTGG |
| BdHsp23.2-M | TGCCCAGCTTCTTCTCAGGTT | CCTTGATCACCAGGCTGTTCT |
| BdHsp21.0-CV | AGTATTGCCTCAGAGCCGAC | TTCTTGGGCACCTTGATCTCC |
| BdHsp40.0-M | CTTTTGTGGTCTTCCCCCGA | AGCAGGTCGAAGTGCTTGAA |
| BdHsfA1a | TCAAACCATAGAGGCAAGCA | ACGCCTGCGAAAACCTTAGA |
| BdHsfA2a | ACATGGTGTGGTACGAGCTG | CAATCTCCTCCCACGGTTCC |
| BdHsfA2b | CACAGTCAAAAGACAGCGGC | CACTTGCGCATGGGTTCATC |
| BdHsfA3a | TTTGGGGCGTGGATCCTTAC | AGAGCAGGGTCGCCAATAAC |
| BdHsfA4a | ATCACCTCCGTCGATGATGC | TCGGAGACCTTAGCTCGTGA |
| BdHsfA5a | ACGAGATCAGCAGGCTCAAG | CAACATTCTCCTGGCGTTGC |
| BdHsfA5b | TTGGGACTTTGTCCCGCAAT | CATCATTTGAGGCACCAGCG |
| BdHsfA6a | CCACAACGCAGAGTGGTTTG | ACTTTCCTCAGTGCTGTCGG |
| BdHsfA6b | ACAATCCTGTCGGAGCCAAG | TGTCATCCTTCGTGCCCTTC |
| BdHsfA6c | ACGTCCAAGCCATGGAAGAG | TGGAAGAACCGTGGGTTACG |
| BdHsfA7a | AACTTCTCCAGCTTCGTCCG | GTGGAGGTTTCCGACGCTTA |
| BdHsfA7b | GCATCGACCAGGGAACTGAA | ACTGACCCGTCAGCAAGAAG |
| BdHsfA8a | ATTTTGTTCAGCCACCGCAC | CAATCAGCAGGGGTGTCAGT |
| BdHsfB1a | TTCGTAAGGTGGTGCCTGAC | TGCCGCAGGATTTAGAGGAC |
| BdHsfB2a | GAGATACACCGCCGGAAGG | CTTCATGTGTCCGAGCTCCC |
| BdHsfB2b | CTGGACCTAATGCCCTCGTG | CCGGTATATCGGCCACGAAT |
| BdHsfB3a | CGACGAGACAAGGCTAGGAG | GCGTTGCACGATTTGATCCT |
| BdHsfB4a | GCAGACTCCGTCAAACCTCA | AGCTCCATCAGCCTACAGGA |
| BdHsfB4b | GTCCAGTAGTCGTACCGCAG | CTATCGTCTAGCCTCACGCC |
| BdHsfB4c | ACCTACGGCTTCAGGAAGGT | TGCATGAAGGGCAAAACGC |
| BdHsfC1a | AACTTCTCCAGCTTCGTCCG | CGCTGTCGATTGTTGCAGAG |
| BdHsfC1b | AGACGTTCCACATGGTCAGC | GCGGAAACCGTAGGTGTTGA |
| BdHsfC2a | TAATCAGCTGGCGGTGGATG | CCTCCGAAACCAAACGAGGA |
| BdHsfC2b | AGCAACAGCTTCGTGGTGAT | ACCATAGGTGTTGAGCTGGC |
