# Supplementary material for: Identification, expression, and functional analysis of Hsf and Hsp20 gene families in Brachypodium distachyon under heat stress
Source: PeerJ. 2021 Oct 1;9:e12267. doi: 10.7717/peerj.12267 (PMC8489411; doi:10.7717/peerj.12267)

**Table S2. Complete DNA sequences of five Hsp20s constructing overexpression vector and the associated maps used in this work.**

| Gene name | CDSsequences |
| --- | --- |
| BdHsp16.9-CI | ATGTCGCTGATTCGCCGTGGCGACGTGTTCGACCCCTTCTCTCTTGATCTCTGGGACCCTTTCTCCTTCGGCTCCGGCAGCGGCAGCATCTTCCCTCGCACCGGCTCCGAGACCGCCAGCTTCGCCGGCGCGCGCATCGACTGGAAGGAGACCCCCGAGGCGCACGTGTTCAAGGCCGACGTGCCGGGGCTGAAGAAGGAGGAGGTGAAGGTGGAGATCGAGGACGGCAACGTGCTGCAGATCAGCGGCGAGCGCAGCAGGGAGCACGAGGAGAAGTCCGACACCTGGCACCGCGTCGAGCGCAGCAGCGGCAAGTTCCTGCGCAGGTTCAGGCTCCCCGACAACGCCAAGACGGAGCAGATCAAGGCGGCCATGGAGAACGGCGTGCTCACGGTTACCGTGCCCAAGGAGGAGGCCAAGAAGACCGATGTCAAGCCAGTTCAGATCACCGGCTAG* |
| BdHsp17.2A-CI | ATGTCGCTGGTGAGGAGGAGCAGCGTGTTCGACCCGCGGTCCGACTTCTGGTTCGACCCCATGGACACCATCGACGGCATCTTCCGCTCCGTCGTCCCGGCGGCCGCCACGGATTCCGACGCCGCCGCCTTCGCCAACGCCCGCATGGACTGGAAGGAGACGCCCGAGGCGCACGTGTTCAAGGCCGACCTCCCGGGAGTGAAGAAGGAGGAGGTGAAGGTGGAGGTGGAAGACGGCAACGTGCTGGTGGTGAGCGGCGAGCGGAGCAAGGAGAAGGAGGACAAGAACGACAAGTGGCACCGCGTCGAGCGCAGCAGCGGCAAGTTCGTCCGCCGCTTCCGTCTGCCGGACAACGCTAAAGTGGAGCAGGTCAAGGCCGGGCTCGAGAACGGCGTGCTCACGGTCACCGTGCCCAAGGCCGAGGTCAAGAAGCCCCAGGTGAAGGCCATCGAGATCTCCGGTTGA* |
| BdHsp17.2B-CI | ATGTCGCTGGTGAGGCGCGGCAGCGTGTTCGACCCATTCTCCCAGGACCTCTGGGACCCGATCGACAGCATCTTCCGCTCCATCGTCCCGGCAGCCGCGGCCTCCTCCGACTTCGACACTGCGGCCTTCGTCAACGCCCGCATGGACTGGAAGGAGACTCCCGAAGCTCACGTGTTCAAGGCGGACCTTCCCGGAGTGAAGAAGGAGGAGGTGAAGGTGGAGGTGGAAGACGGCAACGTGCTGGTGGTGAGCGGCGAGCGCAGCAGGGAGAAGGAGGACAAGAACGACAAGTGGCACCGCGTCGAGCGCAGCAGCGGCAAGTTCGTCAGGCGCTTCCGTTTGCCGGAGAACGCTAAGGTGGAGCAGGTCAAGGCTGGGCTGGAGAACGGCGTGCTCACGGTCACCGTGCCCAAGTCTGAGGTCAAGAAGCCAGAGGTGAAGGCCATCGAGATCTCTGGTTAA* |
| BdHsp18-CII | ATGGAGGGCAGGATGTTCGGGCTGGAGACCCCGCTGATGACGGCGCTGCAGCACCTGCTGGACATCCCCGACGGCGAGTCCACGGGCGGCGTTGGCGGCGGCGCGGGCGGCGTCGAGAAGCAGGGCCCGACGCGTGCCTACGTCCGCGACGCGCGCGCCATGGCGGCCACCCCGGCGGACGTGAAGGAGCTCCCCGGCGCGTACGCGTTCGTGGTGGACATGCCCGGGCTGGGGTCCGGCGACATCAAGGTGCAGGTGGAGGACGAGCGGGTGCTGGTGATCAGCGGCGAGCGGCGCAGGGAGGAGAAGGAGGACGCCAAGTACCTGCGCATGGAGCGCCGCATGGGCAAGTTCATGCGCAAGTTCGTGCTGCCGGAGAACGCCGACATGGACAAGATCTCCGCCGTCTGCCGCGACGGCGTGCTCACCGTCAACGTCGACAAGCTGCCGCCGCCAGAGCCCAAGAAGCCCAAGACCATCAACGTCCAGGTCGCGTGA* |
| BdHsp16.4-CI | ATGTCGCTCGTGAGGCGCAGCGCATTCGACCCCTTCGCCGACTTCTGGGACCCTCTGGACGTGTTCCGCTCCATCGTCCCCGCAGCGTCCGGCAGCGAGACCGCCGCTTTCGCCAACGCCCGTGTGGACTGGAAGGAGACCCCGGAGGCGCACGTGTTCAAGGCCGACCTCCCCGGCGTGAAGAAAGAGGAGGTCAAGGTGGAGGTGGAGGACGGCAACGTGCTCGTGATCAGCGGCGAGCGCAGCAAGGAGAAGGAGGAGAAGAGCGACAAGTGGCACCGCGTGGAGCGCAGCAGCGGGGCGTTCGTGAGGCGGTTCCGCCTGCCGGAGAACGCCAAGGTGGAGCAGGTGAAGGCCGGGCTGGAGAACGGCGTGCTCACGGTCACCGTGCCCAAAGCCGAGGTGAAGAAGCCTGAGGTGAAGGCCATCGAGATCTCTGGCTGA* |


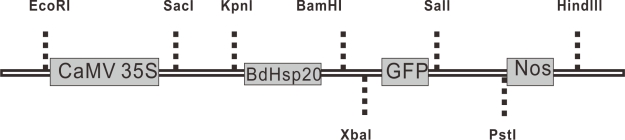

Supplement: Supplemental Information 5 [file peerj-09-12267-s005.doc]
