# Supplementary material for: Identification, expression, and functional analysis of Hsf and Hsp20 gene families in Brachypodium distachyon under heat stress
Source: PeerJ. 2021 Oct 1;9:e12267. doi: 10.7717/peerj.12267 (PMC8489411; doi:10.7717/peerj.12267)
Supplement: Supplemental Information 6 [file peerj-09-12267-s006.docx]

**Table S3 Gene-specific primers used in over-expression plasmid construction**

| Gene name | Primer name | Sequence(5’-3’) | Product size (bp) |
| --- | --- | --- | --- |
| BdHsp16.9-CI | Hsp16.9-CI-p1300-F(BamHI) | 5’- GGggtaccATGTCGCTGATTCGCCGTGG-3’ | 456 |
|  | Hsp16.9-CI-p1300-R(KpnI) | 5’- CGggatccGCCGGTGATCTGAACTGGCTT-3’ |  |
| BdHsp17.2A-CI | Hsp17.2A-CI-p1300-F(BamHI) | 5’- GGggtaccATGTCGCTGGTGAGGAGGAG-3’ | 465 |
|  | Hsp17.2A-CI -p1300-R(KpnI ) | 5’- CGggatccACCGGAGATCTCGATGGCCT-3’ |  |
| BdHsp17.2B-CI | Hsp17.2B-CI-p1300-F(BamHI) | 5’- GGggtaccATGTCGCTGGTGAGGCGCG-3’ | 462 |
|  | Hsp17.2B-CI -p1300-R(KpnI ) | 5’- CGggatccACCAGAGATCTCGATGGCCTT-3’ |  |
| BdHsp18-CII | Hsp18-CII-p1300-F(BamHI) | 5’- GGggtaccATGGAGGGCAGGATGTTCGG-3’ | 498 |
|  | Hsp18-CII-p1300-R(KpnI ) | 5’- CGggatccCGCGACCTGGACGTTGATGG-3’ |  |
| BdHsp16.4-CI | Hsp16.4-CI-p1300-F(BamHI) | 5’- GGggtacc ATGTCGCTCGTGAGGCGCAG-3’ | 444 |
|  | Hsp16.4-CI-p1300-R(KpnI ) | 5’- CGggatcc GCCAGAGATCTCGATGGCCT-3’ |  |
