# Supplementary material for: Identification, expression, and functional analysis of Hsf and Hsp20 gene families in Brachypodium distachyon under heat stress
Source: PeerJ. 2021 Oct 1;9:e12267. doi: 10.7717/peerj.12267 (PMC8489411; doi:10.7717/peerj.12267)
Supplement: Supplemental Information 7 [file peerj-09-12267-s007.docx]

Table S4. List of *Hsp20* and *Hsf* genes in *A. thaliana* and *O. sativa.*

| Gene Name | Locus ID | Gene Name | Locus ID |
| --- | --- | --- | --- |
| AtHsp14.2-CVI | At5g47600 | OsHsp16.0-PX | Os06g14240 |
| AtHsp15.4-CIV | At4g21870 | OsHsp16.6-CI | Os01g04340 |
| AtHsp15.7-PX | At5g37670 | OsHsp16.9A-CI | Os01g04370 |
| AtHsp17.4-CI | At3g46230 | OsHsp16.9B-CI | Os01g04380 |
| AtHsp17.4-CIII | At1g54050 | OsHsp16.9C-CI | Os01g04360 |
| AtHsp17.6A-CI | At1g59860 | OsHsp17.4-CI | Os03g16020 |
| AtHsp17.6B-CI | At2g29500 | OsHsp17.7-CI | Os03g16040 |
| AtHsp17.6C-CI | At1g53540 | OsHsp17.8-CI | Os02g48140 |
| AtHsp17.6-CII | At5g12020 | OsHsp17.9A-CI | Os03g15960 |
| AtHsp17.7-CII | At5g12030 | OsHsp17.9B-CI | Os01g04350 |
| AtHsp17.8-CI | At1g07400 | OsHsp18.0-CI | Os03g16030 |
| AtHsp18.1-CI | At5g59720 | OsHsp18.0-CII | Os01g08860 |
| AtHsp18.5-CI | At2g19310 | OsHsp18.6-CIII | Os02g54140 |
| AtHsp21.7-CV | At5g54660 | OsHsp18.8-CIV | Os07g33350 |
| AtHsp22.0-ER | At4g10250 | OsHsp18.8-ER | Os02g03570 |
| AtHsp23.5-M | At5g51440 | OsHsp19.0-CII | Os02g12610 |
| AtHsp23.6-M | At4g25200 | OsHsp21.8-ER | Os11g13980 |
| AtHsp25.3-CP | At4g27670 | OsHsp22.3-CV | Os05g42120 |
| AtHsp26.5-M | At1g52560 | OsHsp23.2-ER | Os04g36750 |
|  |  | OsHsp23.6-M | Os02g10710 |
|  |  | OsHsp24.0-M | Os02g52150 |
|  |  | OsHsp26.2-M | Os06g11610 |
|  |  | OsHsp26.7-CP | Os03g14180 |
|  |  |  |  |
| AtHsfA1a | At4g17750 | OsHsfA1a | LOC_Os01g39020 |
| AtHsfA1b | At5g16820 | OsHsfA1b | LOC_Os06g36930 |
| AtHsfA1d | At1g32330 | OsHsfA1c | LOC_Os03g63750 |
| AtHsfA1e | At3g02990 | OsHsfA3a | LOC_Os02g32590 |
| AtHsfA2a | At2g26150 | OsHsfA4a | LOC_Os01g54550 |
| AtHsfA3a | At5g03720 | OsHsfA4b | LOC_Os05g45410 |
| AtHsfA4a | At4g18880 | OsHsfA5a | LOC_Os02g29340 |
| AtHsfA4c | At5g45710 | OsHsfA6a | LOC_Os03g06630 |
| AtHsfA5a | At4g13980 | OsHsfA6b | LOC_Os03g53340 |
| AtHsfA6a | At5g43840 | OsHsfA6c | LOC_Os03g58160 |
| AtHsfA6b | At3g22830 | OsHsfA6d | LOC_Os07g08140 |
| AtHsfA7a | At3g51910 | OsHsfA6e | LOC_Os10g28340 |
| AtHsfA7b | At3g63350 | OsHsfA8a | LOC_Os03g12370 |
| AtHsfA8a | At1g67970 | OsHsfB1a | LOC_Os08g36700 |
| AtHsfA9a | At5g54070 | OsHsfB1b | LOC_Os09g28200 |
| AtHsfB1a | At4g36990 | OsHsfB2a | LOC_Os08g43334 |
| AtHsfB2a | At5g62020 | OsHsfB2b | LOC_Os09g35790 |
| AtHsfB2b | At4g11660 | OsHsfB3a | LOC_Os04g48030 |
| AtHsfB3a | At2g41690 | OsHsfB3b | LOC_Os09g28354 |
| AtHsfB4a | At1g46264 | OsHsfB4a | LOC_Os03g25120 |
| AtHsfCa | At3g24520 | OsHsfB4b | LOC_Os07g44690 |
|  |  | OsHsfC1a | LOC_Os01g43590 |
|  |  | OsHsfC1b | LOC_Os01g53220 |
|  |  | OsHsfC2a | LOC_Os02g13800 |
|  |  | OsHsfC2b | LOC_Os06g35960 |
